# Metamaterial-based transmit and receive system for whole-body magnetic resonance imaging at ultra-high magnetic fields

**DOI:** 10.1371/journal.pone.0191719

**Published:** 2018-01-25

**Authors:** Tim Herrmann, Thorsten Liebig, Johannes Mallow, Christian Bruns, Jörg Stadler, Judith Mylius, Michael Brosch, Jan Taro Svedja, Zhichao Chen, Andreas Rennings, Henning Scheich, Markus Plaumann, Marcus J. B. Hauser, Johannes Bernarding, Daniel Erni

**Affiliations:** 1 Institute of Biometrics and Medical Informatics, Otto-von-Guericke University Magdeburg, Magdeburg, Germany; 2 General and Theoretical Electrical Engineering (ATE), University of Duisburg-Essen, and CENIDE–Center for Nanointegration Duisburg-Essen, Duisburg, Germany; 3 Leibniz Institute for Neurobiology (LIN), Magdeburg, Germany; 4 Center for Behavioral Brain Sciences, Magdeburg, Germany; Linköping University, SWEDEN

## Abstract

Magnetic resonance imaging (MRI) at ultra-high fields (UHF), such as 7 T, provides an enhanced signal-to-noise ratio and has led to unprecedented high-resolution anatomic images and brain activation maps. Although a variety of radio frequency (RF) coil architectures have been developed for imaging at UHF conditions, they usually are specialized for small volumes of interests (VoI). So far, whole-body coil resonators are not available for commercial UHF human whole-body MRI systems. The goal of the present study was the development and validation of a transmit and receive system for large VoIs that operates at a 7 T human whole-body MRI system. A Metamaterial Ring Antenna System (MRAS) consisting of several ring antennas was developed, since it allows for the imaging of extended VoIs. Furthermore, the MRAS not only requires lower intensities of the irradiated RF energy, but also provides a more confined and focused injection of excitation energy on selected body parts. The MRAS consisted of several antennas with 50 cm inner diameter, 10 cm width and 0.5 cm depth. The position of the rings was freely adjustable. Conformal resonant right-/left-handed metamaterial was used for each ring antenna with two quadrature feeding ports for RF power. The system was successfully implemented and demonstrated with both a silicone oil and a water-NaCl-isopropanol phantom as well as in vivo by acquiring whole-body images of a crab-eating macaque. The potential for future neuroimaging applications was demonstrated by the acquired high-resolution anatomic images of the macaque’s head. Phantom and in vivo measurements of crab-eating macaques provided high-resolution images with large VoIs up to 40 cm in xy-direction and 45 cm in z-direction. The results of this work demonstrate the feasibility of the MRAS system for UHF MRI as proof of principle. The MRAS shows a substantial potential for MR imaging of larger volumes at 7 T UHF. This new technique may provide new diagnostic potential in spatially extended pathologies such as searching for spread-out tumor metastases or monitoring systemic inflammatory processes.

## Introduction

Human whole-body magnetic resonance imaging (MRI) systems with a static magnetic flux density field (B_0_ field) of either 1.5 T or 3 T are the workhorses of routine clinical applications [1–5]. During the last decade, a trend towards performing MRI at higher magnetic flux densities (B_0_) is observed, since higher B_0_ fields lead to both an improved signal-to-noise ratio (SNR) and an enhanced spatial resolution, thus providing for a more sensitive detection. Currently, these features of ultra-high field (UHF) MRI are especially exploited in functional MRI studies, where the brain activity is investigated. Recently, the certification process of human whole-body 7 T MRI systems for clinical diagnostics has been started. It is to be expected that clinical 7 T MRI examinations will become more frequent in near future. Although substantial progress has been made since the introduction of 7 T MR scanners in research some problems still remain and call for new solutions. In MRI performed at magnetic flux densities of up to 3 T, usually a body coil irradiates the required excitation energy (B_1_^+^ field) into the object, whereas dedicated, smaller radio frequency (RF) coils are used for detection of the MR signals, which are ultimately transformed into the well-known MR images.

**Fig 1:**
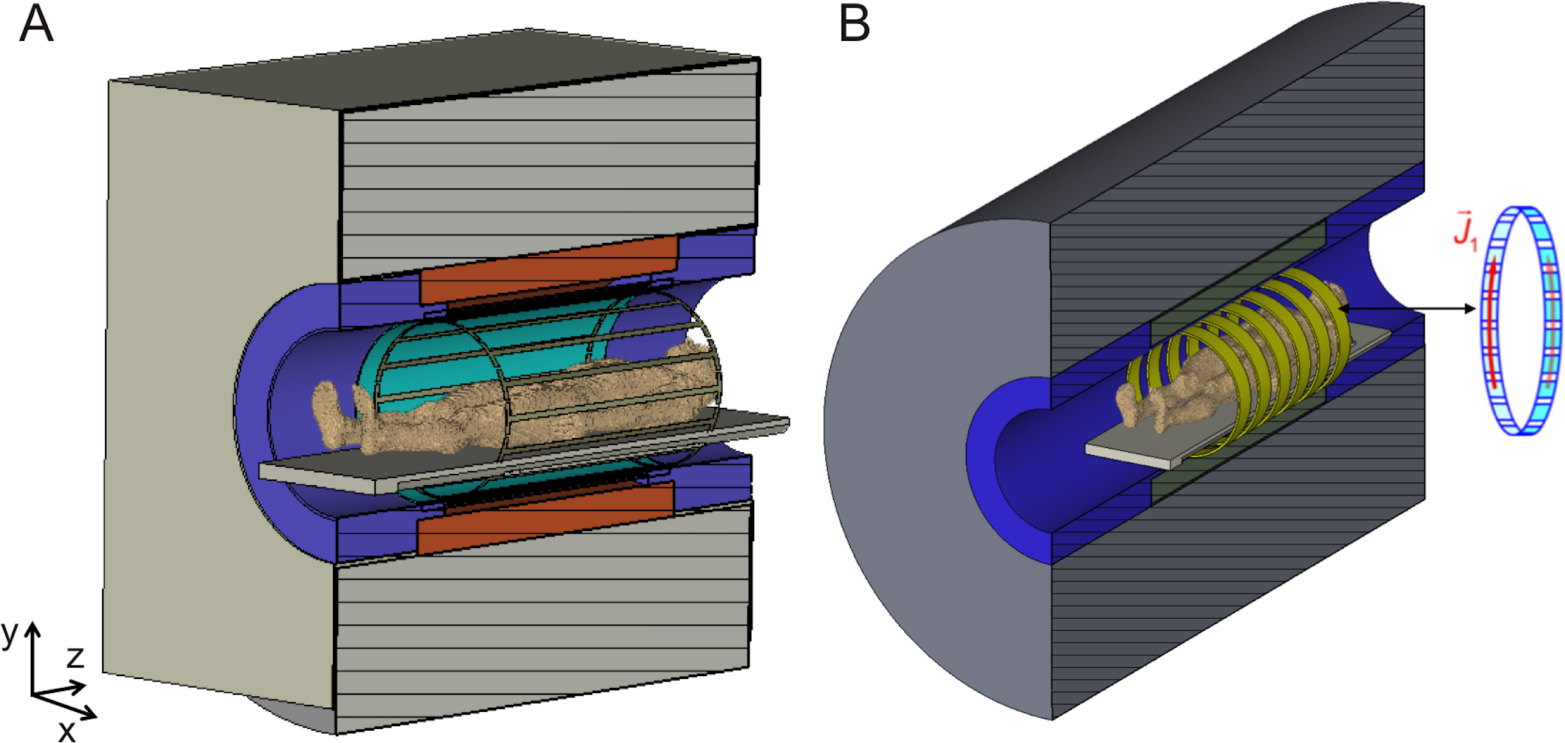
Operating principles of a traditional MRI system and of the composite right-/left-handed (CRLH) Metamaterial Ring Antenna System (MRAS). (A) A traditional body coil resonator in a birdcage low pass architecture. The coils form a standing radio-frequency field inside the biological object. (B) Sketch of an ideal experimental setup for whole-body imaging with excitation and detection performed by a CRLH MRAS fully integrated in human whole-body UHF MRI system including the patient table. Each ring antenna can act as a transmit or receive unit. $\vec{J_{1}}$ is the current density.

The electromagnetic characteristic of body coils is known to depend on the resonant frequency, which is linearly dependent on the magnetic field strength. This was shown by Vaughn et al. where a transverse electromagnetic (TEM) body coil was developed and demonstrated at a 7 T MRI system [6] with the outcome of low efficiency and interference effects in the transverse plane. At lower field strengths body coils act as resistively loaded resonators that conduct the RF energy into the body [7]. By contrast, at higher magnetic field strengths (7 T and beyond), RF coils tend to behave like antennas. In the latter, the feeding power is spread over the surface of the body of the subject, from where it propagates into the inner parts of the body [8], thus mimicking radiation behavior. Usually, the body coil of standard clinical MRI systems is a large RF coil in birdcage architecture [9] that transmits the excitation energy in a highly homogeneous and efficient manner (Fig 1A). Systems equipped with such coils may provide large fields of view (FoV), even though smaller receive coils are typically used. Whole-body imaging is then achieved by moving the patient slowly through this optimal volume, i.e., using a moving table [10,11].

During the last decade MRI systems with static B_0_ fields of 7 T or more [12] were introduced in research in order to increase the SNR and spatial resolution in functional MRI (fMRI) [13,14] and anatomic studies as well as in other promising areas [15]. However, application were mainly restricted to small or medium-sized volumes of interests (VoI) as at ultra-high magnetic fields, the higher resolution and the enhanced SNR come with a decreased wavelength of the excitation field (B_1_^+^) which is often as small or even smaller as the dimensions of the examined object. In this case, severe B_1_^+^ field inhomogeneities may occur [16] due to standing wave effects. Furthermore, at UHF MRI the RF energy absorbed by the investigated body part is considerably higher when compared to MRI systems operating at lower B_0_ fields. So far, the vendor-equipped UHF MRI systems support imaging of VoIs with dimensions determined by the characteristics of the transmit (Tx) and receive (Rx) coils. At UHF MRI volume or multi-element transmit coils are being employed for investigating the brain, [17] heart, [18] or knee [19]. The limitation to smaller VoIs is also reflected by the fact that body coils or arrays are not yet available for commercial 7 T (or higher) whole-body MRI.

During the last years, however, efforts have been undertaken to develop concepts and RF coils that make larger VoIs accessible for UHF MRI. A step in this direction is the so-called travelling-wave (TW) excitation approach that makes use of the RF shield as part of the gradient system in combination with a simple patch antenna [20]. This approach yields a sufficiently uniform B_1_^+^ field distribution for the imaging of a human extremity (leg) [20] or for neuroimaging of smaller non-human primates [21], thus enabling the imaging at VoIs of intermediate size. Unfortunately, the B_1_^+^ transmit efficiency, i.e., the required RF energy to generate the B_1_^+^ field is lower than that of both the body coils conventionally used in clinical 3 T MRI systems [22] and of the UHF body-part volume resonators of birdcage architecture [23,24]. Usually, birdcage volume coils confine the RF energy in the structure enclosed by the RF coil.

The acquisition of whole-body images at UHF MRI requires the control of the RF energy introduced into the examined body region. This calls for novel conceptual solutions, like the one provided by the theory of conformal resonant right-/left-handed (CRLH) metamaterial ring antennas [25], which relies on the excitation of the fundamental transverse electric circular waveguide mode (TE_11_) in the MRI scanner bore by an adaptive RF antenna system. This concept is briefly summarized in the next section. Whereas the theoretical foundations have been recently put forward [25] and examined in field simulations [26] no experimental realization or even an *in vivo* examination of a whole-body transmit system based on CRLH metamaterial ring antennas has been reported so far (Fig 1B).

In the present paper we describe the first experimental study of a potential whole-body imaging system based on CRLH metamaterial ring antennas. The rationale of our study is the following: We first show the design of CRLH metamaterial ring antennas and unravel the detailed functionalities of the developed metamaterial ring antenna system (MRAS). Than we show that CRLH metamaterial ring antennas are indeed appropriate for the construction and operation of a whole-body imaging system run in an UHF MRI system. Characteristic functionalities of the MRAS will be investigated in experiments, namely its high SNR, its ability to image large VoIs, as well as its efficient use of RF energy. To these purposes three experimental ring antenna setups were built, which consist of (1) a MRAS for measuring phantoms, (2) a MRAS for whole-body image acquisition of a macaque, and (3) a MRAS to allow high-resolution imaging of the head of a macaque. These setups differ in the geometric and functional arrangements of their ring antennas. We demonstrate that MRASs allow *in vivo* 7 T imaging of VoIs that are 4–6 times larger than that of standard 7 T imaging capability. The MRAS concept is also different from the standard volume body coil concept with its fixed architecture as the rings can be positioned and combined in a flexible way allowing different experimental setups. This will be shown in the present work and the outcome of these three experimental ring antenna setups will give an overview of how many ring antennas the Metamaterial Ring Antenna System should consists of to provide a high SNR and B_1_^+^ transmit efficiency (Fig 1B).

This new concept may also be of interest for diagnostic imaging as recently, the certification process of introducing human whole-body 7 T MRI systems into clinical routine has been started. Therefore, clinical 7 T MRI examinations will become more frequent in near future and the presented concept may support additional capabilities to use 7 T MRI for new diagnostic techniques such as whole-body imaging at UHF.

## Materials and methods

### Metamaterial ring antennas

The CRLH metamaterial ring antennas where build in-house. They were designed to support a full wavelength resonance pattern along the circumference of the ring structure to match the surface-current distribution of the excited TE_11_ mode.

**Fig 2:**
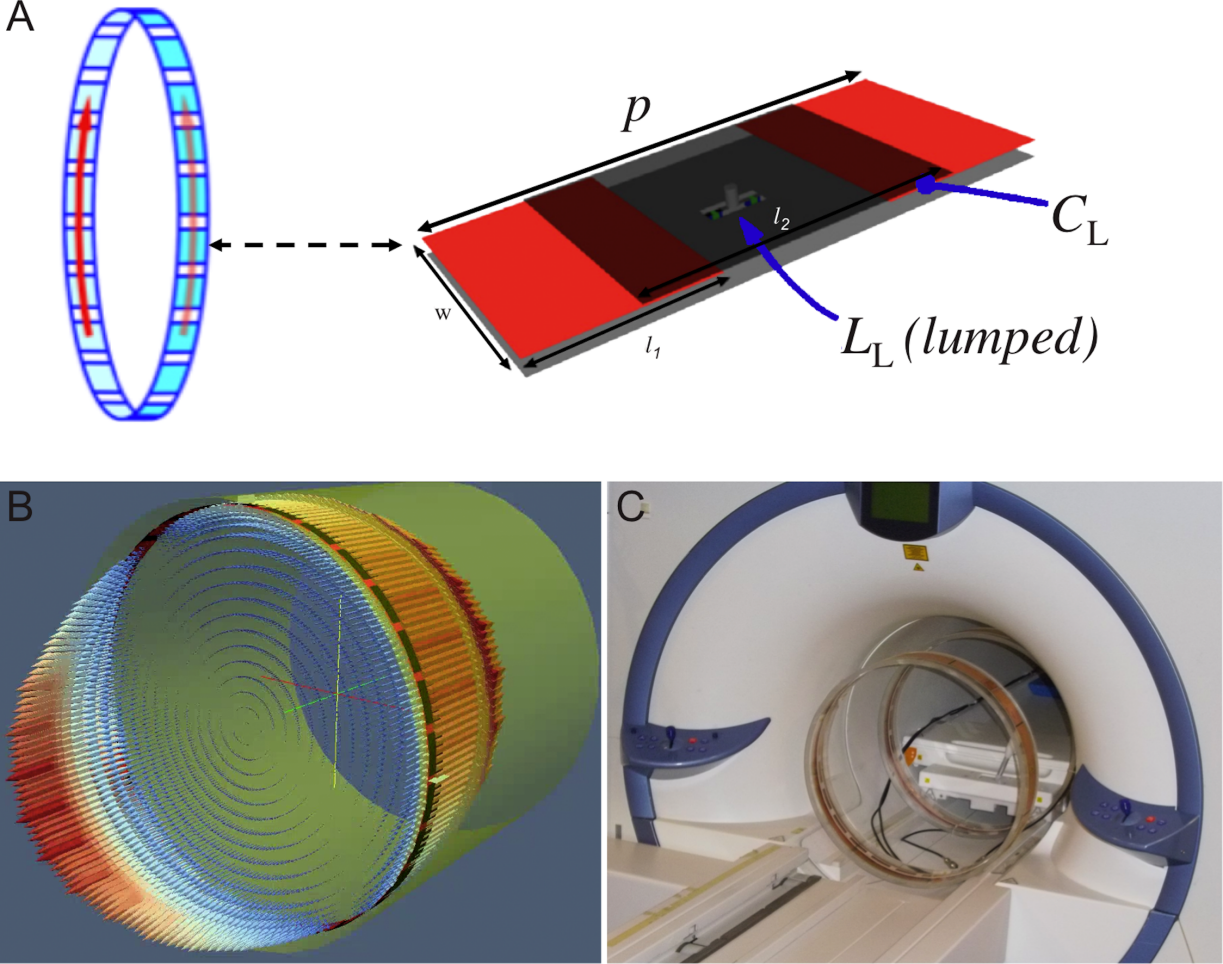
Design and installation of the metamaterial ring antennas. (A) Schematic sketch of a unit cell of the metamaterial ring antenna design. The quantity *p* stands for the footprint (i.e. periodicity) of one unit cell. Metal-insulator-metal (MIM) multilayer realization of the unit cell (outer, intermediate and inner metal layers are displayed in black, red and grey) where the shunt inductor *L*_L_ is introduced as a lumped (or chip) element and *C*_L_ as the capacity for the left-handed contribution. (B) Simulation model of the metamaterial ring antenna with the current density $\vec{J_{1}}$ on the surface along the ring structure and the H field distribution. (C) Exemplary installation of one pair of mobile and versatile metamaterial ring antennas (diameter 51 cm) inside of the 7 T MRI system bore (diameter 60 cm).

The whole diameter of all three constructed ring antenna was 51 cm for a mobile versatile installation inside the 7 T MRI system bore (diameter = 60 cm) because an installation behind the MR bore housing wasn’t possible. Each ring structure is formed by three (outer / intermediate / inner) metal layers, which encompass a spacing of 1.28 mm followed by 10 mm, where the outer and intermediate layer are part of a Rogers 3010 substrate (ε_r_ = 10.2). The inner metal layer corresponds to an acrylic glass ring (inner diameter 50 cm) acting as the overall carrier. The resulting CRLH ring antenna has 24 unit cells with a length *p* = 62.83 mm and a width w = 11 mm, with a gap width of 13.83 mm (Fig 2A). The resulting length of the metal patch on the inner layer is therefore 49.00 mm. The CRLH metamaterial ring antenna is based on a periodic arrangement of such elongated metal patches on both sides of the Rogers substrate, namely on the inner and intermediate metal layer, where the intermediate periodic structure is shifted by *p*/2 against the inner layer structure. A 50 mm wide metal ring acts as ground plane on the outer layer and completes the antenna topology. This ground plane ring is actually divided into 8 sections that are equidistantly distributed along the circumference. These sections are capacitively coupled and thus best suited to inhibit eddy currents stemming from field gradient switching.

### Metamaterial Ring Antenna System

Periodic material arrangements whose structural properties are designed to modulate electromagnetic (EM) waves in such a way that desired surface-current density distributions can be tailored are known as electromagnetic metamaterials. Their structural features have both inductive and capacitive characteristics. Components made of metamaterials are small, their dimensions typically being shorter than the wavelengths of the RF field of the UHF MRI system. Therefore, a suitable choice of both the metamaterial and of the geometry of the components made of it yields optimal and adaptable surface-current density distributions, which can be tailored to mimic the surface-current distribution of the targeted travelling-wave mode. Hence, any desired excitation of B_1_^+^ mode fields may be generated by a suitable experimental design [27].

The test setup of our MRAS is based on a set of conformal resonant right-/left-handed ring antennas [25]. The MRAS is composed of at least one pair of ring antennas, but more ring antennas can be added easily. The rings may act as transmitters and receivers, but also as reflectors which is a function usually not required in standard body coils at lower fields. When working as reflector, the ring antenna must be short-circuited. The MRAS utilizes the RF shield as a circular waveguide, where waveguide modes are excited at any desired position depending on the number, function, and position of the ring antennas installed in the MRI system bore. Depending on the ring antenna configuration either travelling or standing EM waves can be formed. The optimal surface-current density distribution closely matches the surface-current of the fundamental transverse electric TE_11_ mode of a circular waveguide represented by the RF shield at the given diameter (Fig 2B). Hence, when using quadrature feeding of the RF energy into the antenna, any of the ring antennas contributes to the excitation of a propagating, circularly polarized (CP) B_1_^+^ field.

### Experimental setups with Metamaterial Ring Antenna System

In our experiments up to three ring antennas (diameter = 51 cm) were placed into the MRI system bore (inner diameter = 60 cm). The insertion of the ring antennas prevents the operation of the patient table (Fig 2C). As a consequence whole-body imaging of an adult human with all experimental setups was not allowed at this time. To cope with this restriction, we have performed 3 sets of proof of principle experiments, which were designed to study different key features of MRAS. In conjunction, these 3 experimental ring antenna setups demonstrate that MRAS systems are well suitable for whole-body imaging.

**Fig 3:**
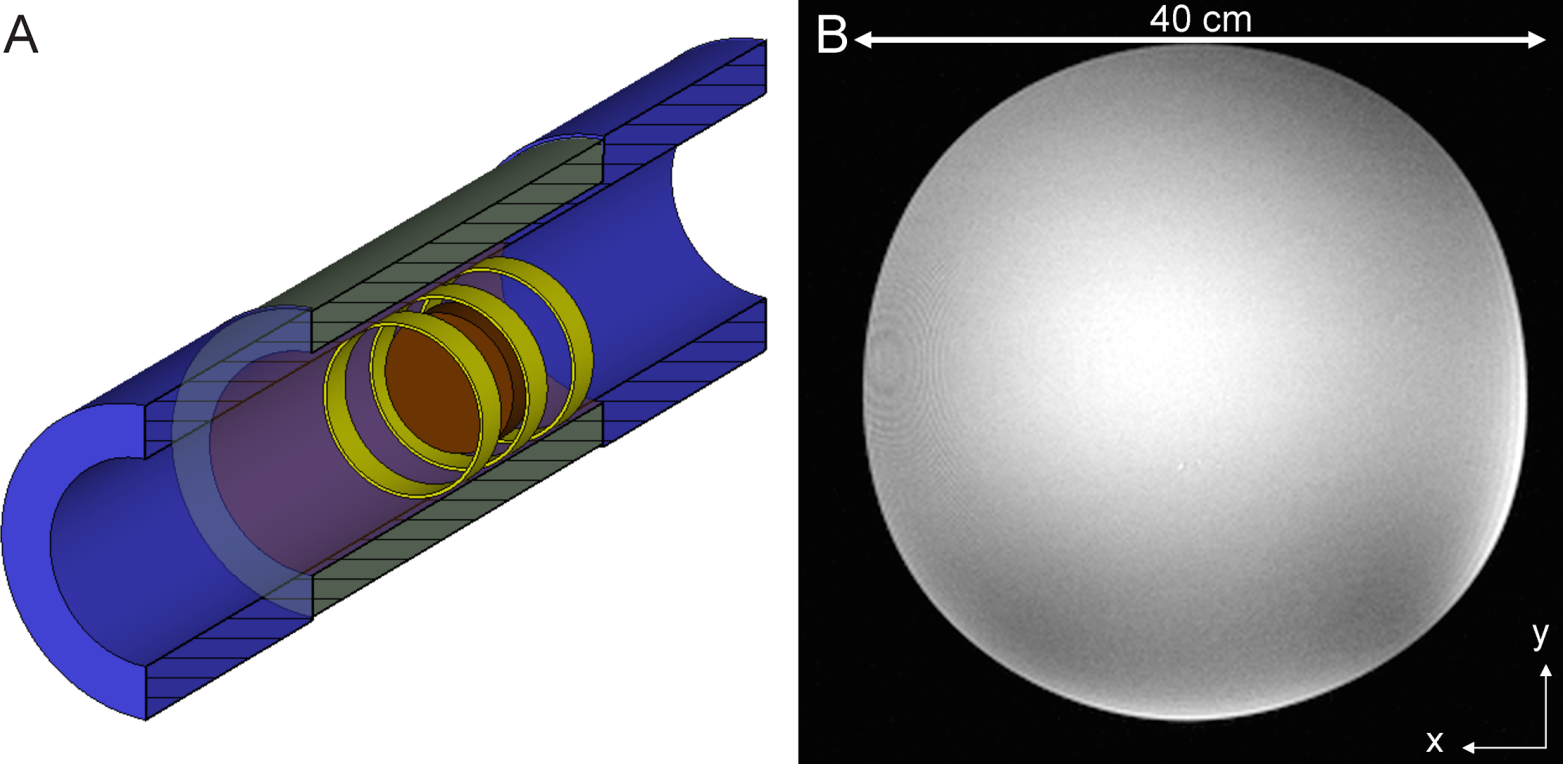
Exemplary images of a large phantom acquired with a Metamaterial Ring Antenna System. (A) Sketch of the first experimental setup with three ring antennas inside the bore of the MRI system without patient table. A cylindrical silicone oil phantom of 40 cm diameter and 12 cm length is placed in the center of the MRAS (image plane perpendicular to the cylinder axis). The silicone oil has a low permittivity of ε_r_ = 2.3. (B) Image of the cylindrical silicone oil phantom acquired in a 7 T MRI system equipped with a MRAS. The distortion of the image is due to insufficient linearity of the Siemens SC72d gradient system when imaging extended objects with more than 20 cm in x-y-direction.

The first experimental ring antenna setup consists of three ring antennas (Fig 3A). Two ring antennas were placed at the end positions of the MRAS, and one ring was placed in the middle position. The end rings served for both transmit and receive whereas the central ring was used for receive only. A cylindrical silicone oil phantom of 40 cm diameter and 12 cm length was used as load. These experiments were performed to test the SNR characteristics of the MRAS in a large homogeneous VoI (silicone oil has low permittivity of ε_r_ = 2.3 which leads to an almost undistorted B_1_^+^ field distribution and thus images free of interference artifacts). In addition, the MR images of the phantom also allowed assessing the quality of the excited fundamental transverse electric circular waveguide mode (TE_11_) under low dielectric load. For comparison of the MRAS sensitivity, the SNR was measured in a selected image dataset. The signal values of each two-port ring antenna were sum-of-squares combined into a whole image dataset. The mean value of the signal intensity in a selected ROI_signal_ in the center of the phantom and a second ROI_noise_ in a signal free area outside of the phantom was measured to form the quotient of both. For calculation of the SNR the associated background-noise coil-element correction factors for this mean value approach have to be applied by multiplication with the ROI_signal_/ROI_noise_ ratio [28,29].

**Fig 4:**
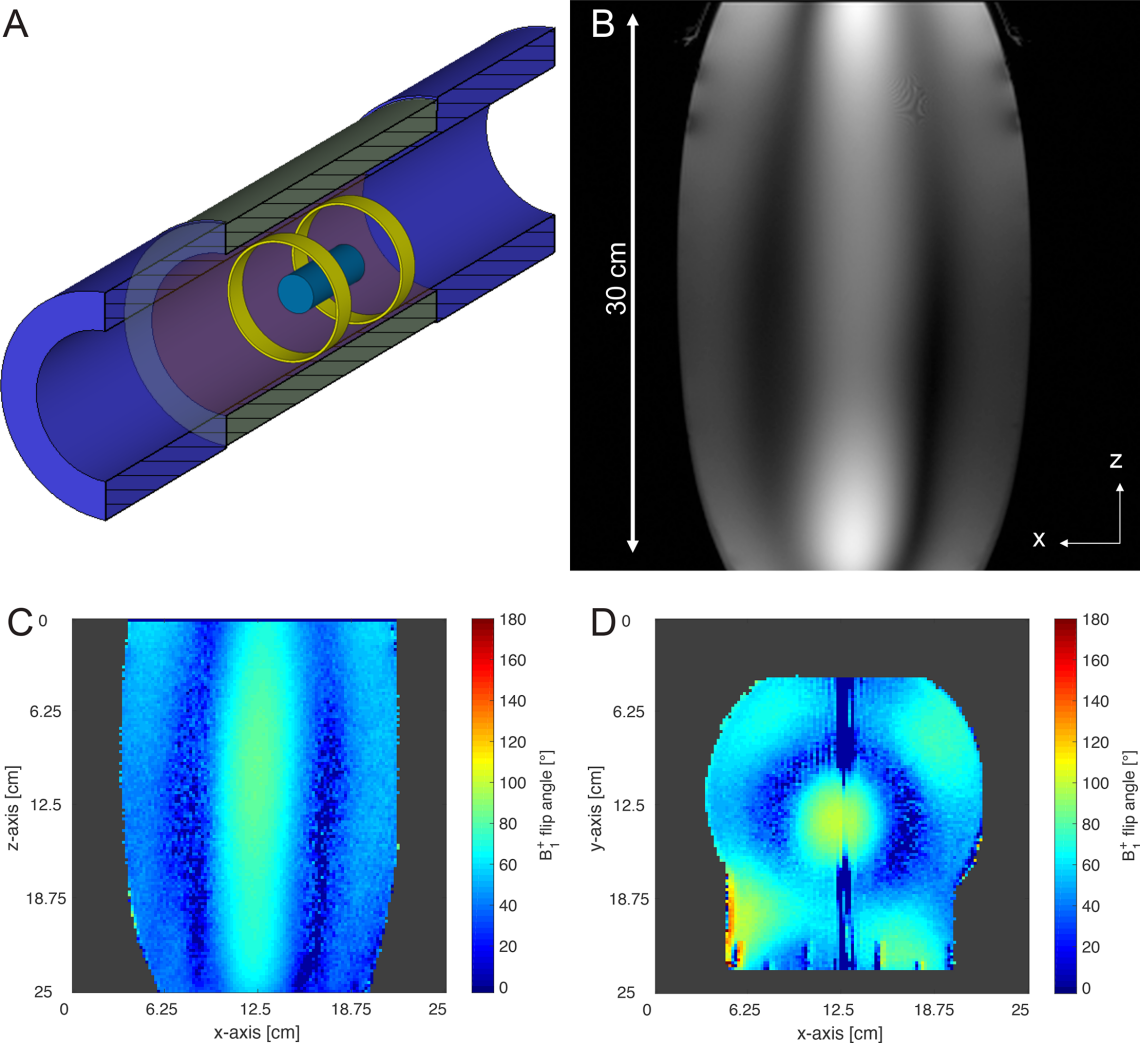
Metamaterial Ring Antenna System with a large water-NaCl-isopropanol phantom as sample for imaging. (A) Sketch of the experimental setup with two ring antennas inside the bore of the MRI system without patient table. A cylindrical water-NaCl-isopropanol phantom of 20 cm diameter and 40 cm length is placed in the center of the MRAS. The water-NaCl-isopropanol solution has a permittivity of ε_r_ = 58.2 and σ = 0.92 S/m. (B) Image of the cylindrical water-NaCl-isopropanol phantom acquired in a 7 T MRI system equipped with a MRAS. The distortion is due to insufficient linearity of the Siemens SC72d gradient system when imaging extended objects with more than 30 cm in z-direction. (C) B_1_^+^ flip angle map at the center of the water-NaCl-isopropanol phantom in transversal direction. The background noise of this result was cropped. (D) B_1_^+^ flip angle map around the center of the water-NaCl-isopropanol phantom in coronal direction. The background noise of this result was cropped. (see S1 File).

**Fig 5:**
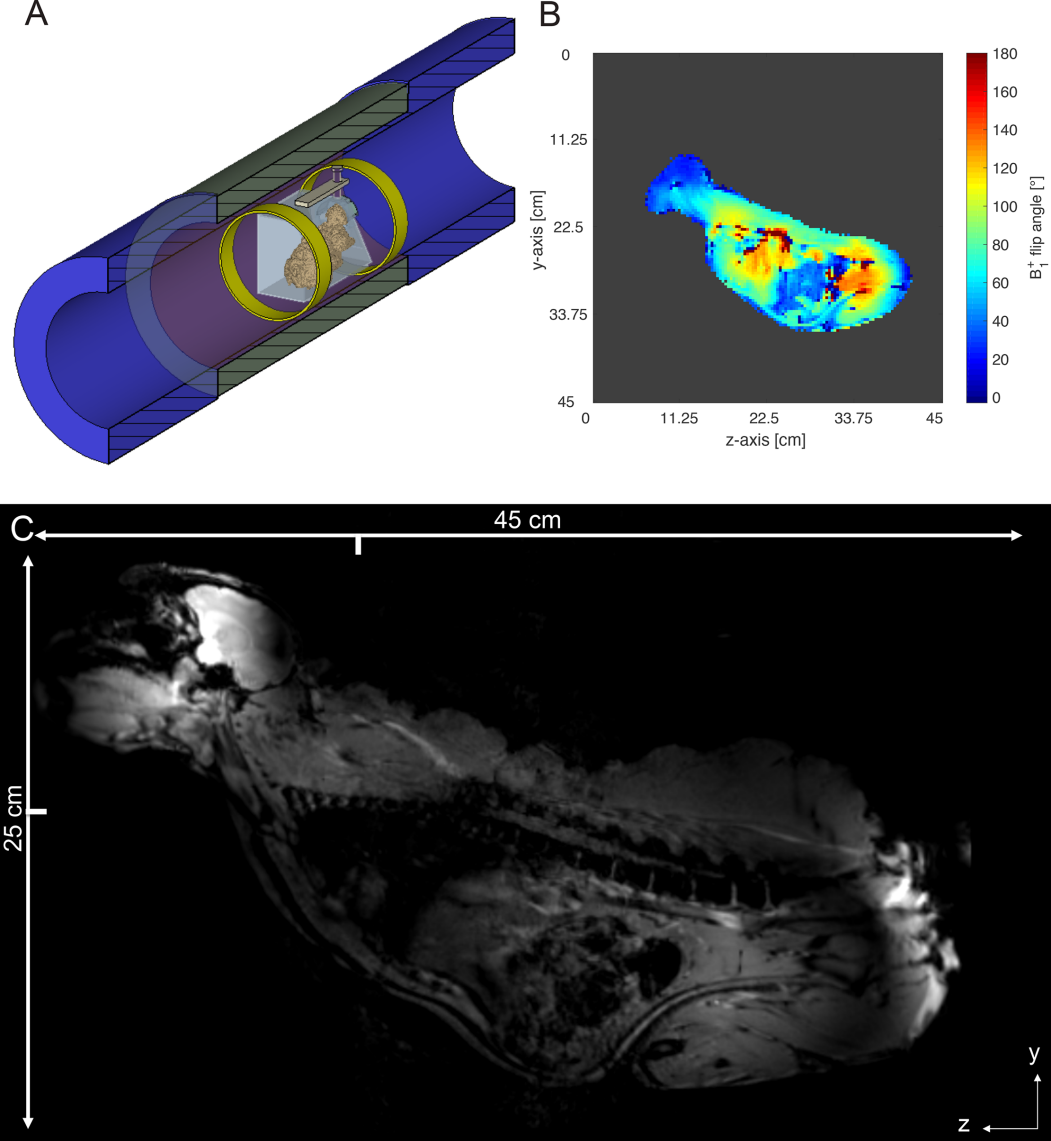
Whole-body *in vivo* imaging of a crab-eating macaque. (A) Sketch of the second experimental setup: the animal-fixation device with two ring antennas both used for transmit and receive. (B) B_1_^+^ flip angle map of the whole crab-eating macaque. The background noise of this result was cropped. (C) *In vivo* image of the whole crab-eating macaque acquired by the two ring antenna MRAS for whole-body MRI and an optimized 3-element phased array monkey head coil for receive only. The ticks indicate the y and z position of the iso-center of the MRI system. (see S1 File).

The second experimental ring antenna setup should investigate the capability of MRAS to image larger (in z-direction) VoIs and objects with higher permittivity as typical for *in vivo* studies. We (1) performed experiments on a cylindrical water phantom that contained water (53%), isopropanol (45%) and sodium chloride (2%) (diameter = 20 cm, length = 40 cm) (Fig 4A), and (2) acquired whole-body images of an adult female crab-eating macaque (Fig 5A) [30]. The higher permittivity (ε_r_ = 58.2) and conductivity (σ = 0.92 S/m) of the water-NaCl-isopropanol solution may lead to stronger distorted B_1_^+^ field distributions, which usually yield to more realistic images with typical interference artifacts. The material parameters represent averaged parameters to mimic the average properties of a human torso based on human tissue material parameters [31]. The choice to select a macaque, which is about 45 cm tall, as an *in vivo* subject for imaging is motivated by the goal to acquire data from complex biological structures under the above-mentioned spatial restrictions of the MRAS-equipped bore of the MRI system. A second rationale was to provide the neuroscience research group of our site working with macaques monkeys [21,24,32] with a new and potentially better data acquisition concept. For the experiments with the water-NaCl-isopropanol phantom and crab-eating macaque, the MRAS was configured as a two ring antenna systems both for transmit and receive which were placed at the end positions of the VoI (Fig 5A). This configuration was necessary, as the fixation system for the macaque monkey did not allow positioning any additional ring antenna closer to the animal.

**Fig 6:**
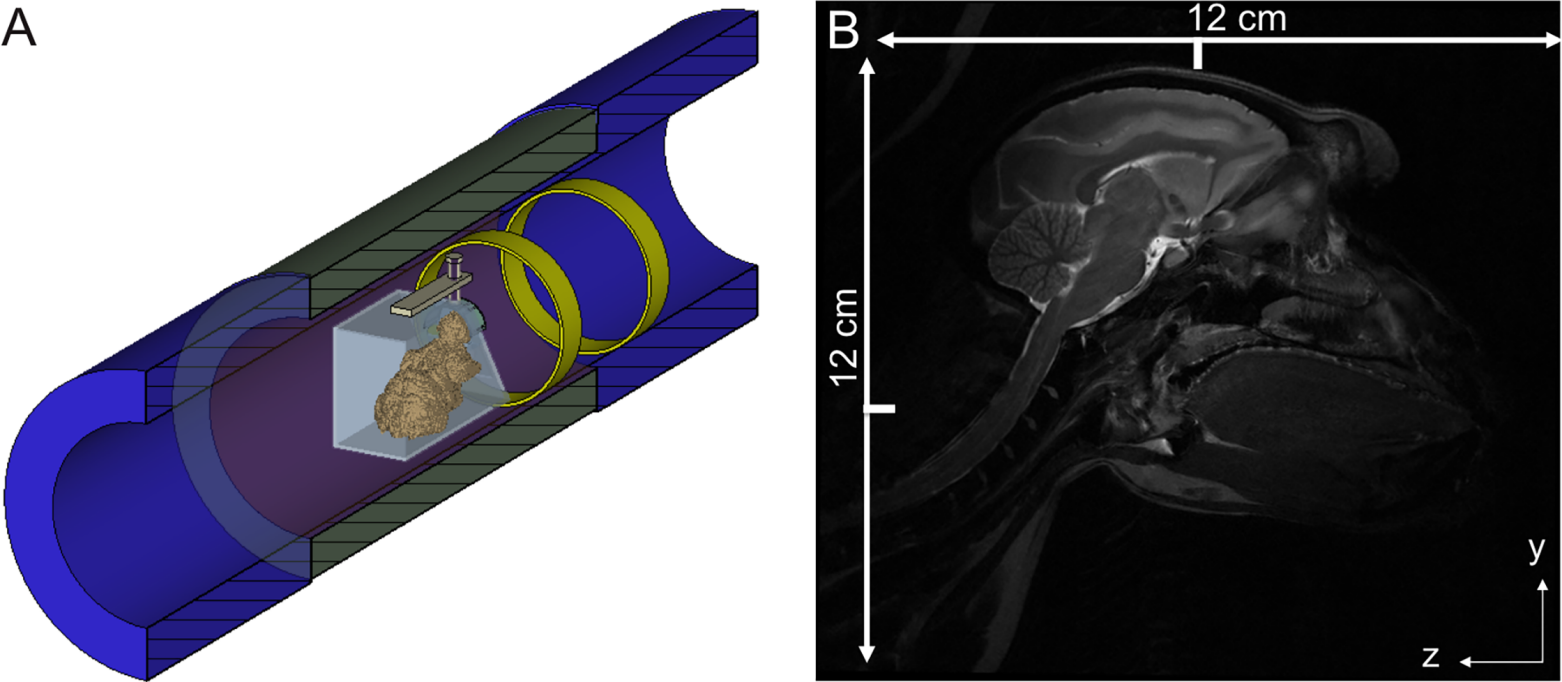
High-resolution *in vivo* imaging of the macaque’s head. (A) Sketch of the experimental setup with one ring antenna for transmit and an additional ring antenna acting as a wave reflector for improving the directivity of the electromagnetic waves for head imaging when using the 3-element, phased-array head receive coil. (B) *In vivo* MR image acquired with the system depicted in Fig 6A. The high-resolution images show a detailed view of the main parts of the crab-eating macaque brain (gray and white matter, cerebellum, and hippocampus). In the upper left corner a small back-folding artifact is visible which does not affect the imaging of the brain parts. The ticks indicate the distance to the y and z position of the iso-center of the MRI system.

A third experimental ring antenna setup examined the ability of MRAS to increase the B_1_^+^ transmit efficiency and the SNR for high-resolution imaging. For this purpose, the bore of the MRI system was equipped with one ring antenna for transmit whereas the remaining ring antenna served as an idle-running reflector (Fig 6A). Note that the two antennas were placed at the same side of the subject. Whereas the ring antenna close to the macaque acted as the transmitter, the more distant ring reflected the EM waves back towards the subject. This arrangement of antennas allowed an increase in the B_1_^+^ field directivity toward the object. The smaller receive coil was placed over the head of the subject. For *in vivo* high-resolution brain imaging, again, adult female crab-eating macaque was used. This setup should maximize the B_1_^+^ transmit efficiency of the MRAS and provided good SNR with the phased array coil. All experimental ring antenna setup graphics were created with the field simulation software CST Microwave Studio 2016 (CST GmbH, Darmstadt, Germany).

### In vivo experiments

The *in vivo* experiments of adult female crab-eating macaques (*Macaca fascicularis*; age 6 years, weight 7–8 kg) were approved by the Authority for Animal Care and Ethics (Landesverwaltungsamt) of the federal State of Sachsen-Anhalt (No. 28-42502-2-1129 IfN) and conformed to the rules for animal experimentation of the European Communities Council Directive (2010/63/EU). During MRI measurements, the monkey was kept under general anesthesia with a mixture of ketamine (2mg/kg) and xylazine (5 mg/kg). The monkey was held in the sphinx position with a fixation device for the head developed in-house.

### MRI systems

The investigated mobile versatile metamaterial-ring antennas were inserted into the bore of a human whole-body 7 T UHF MRI research system (Magnetom 7 T, Siemens Healthcare GmbH, Erlangen, Germany), with inner bore diameter of 60 cm. The 7 T MRI system was equipped with a SC72d gradient system with a maximum amplitude of 70 mT/m and maximum slew rate of 200 mT/m/ms. The gradient system was equipped with a 147 cm long RF shield with an inner diameter of 64 cm. The system control software version was VB17 (UHF). The linear pulse power amplifier (LPPA 13080W, COMET Stolberg, Stolberg, Germany) providing the RF power was equipped with eight 1 kW RF power amplifiers combined with a power combiner and generates 8 kW (excluding the transmissions losses).

Furthermore, a human whole-body 3 T MRI system (Magnetom Trio 3 T, Siemens Healthcare GmbH, Erlangen, Germany) was used for body coil comparison SNR measurements. The 3 T MRI system was equipped with a gradient system with a maximum amplitude of 40 mT/m and maximum slew rate of 200 mT/m/ms. The linear pulse power amplifier provides an amount of 35 kW (excluding the transmissions losses) RF power.

### MRI sequences

To obtain the B_1_^+^ transmit efficiency and the B_1_^+^ field distribution a turbo Fast Low-Angle Shot (FLASH) B_1_^+^ flip angle mapping sequence [33] based on a turbo FLASH sequence preceded by a magnetization preparation (Siemens work-in-progress package, provided by Hans-Peter Fautz) was used. The turbo FLASH B_1_^+^ flip angle mapping sequence was used with the following parameter settings for the silicone oil phantom: time of echo (TE) = 1.83 ms, time of repetition (TR) = 6000 ms, resolution = 3.125×3.125×8 mm^3^, flip angle = 85°, rectangular pre-saturation pulse duration = 1.5 ms. For the water-NaCl-isopropanol phantom: TE = 1.83 ms, TR = 5000 ms, resolution = 1.95×1.95×8 mm^3^, flip angle = 85°, rectangular pre-saturation pulse duration = 1.5 ms (Fig 4C and 4D). For the crab-eating macaques: TE = 1.83 ms, TR = 5000 ms, resolution = 3.35×3.35×8 mm^3^, flip angle = 85°, rectangular pre-saturation pulse duration = 1.5 ms (Fig 5B).

Images of the cylindrical silicone oil phantom were acquired with a 2D gradient-echo (GRE) sequence with the following parameter settings: TE = 4.23 ms, TR = 113 ms, flip angle = 45°, resolution = 1.75×1.75×2.5 mm^3^. Images of the cylindrical water-NaCl-isopropanol phantom were acquired with a 2D gradient-echo (GRE) sequence with the following parameter settings: TE = 3.69 ms, TR = 200 ms, flip angle = 60°, resolution = 1.17×1.17×8 mm^3^. For whole-body MRI of the macaque monkey (Fig 5C) a 2D FLASH sequence was used with the following parameter settings: TE = 4 ms, TR = 435 ms, flip angle = 45°, resolution = 0.87×0.87×2 mm^3^. In the third experimental setup used for image the monkey brain, images were acquired using a 2D Turbo Spin Echo (TSE) sequence with the following parameter settings: TE = 68 ms, TR = 12220 ms, resolution = 0.31×0.31×1.5 mm^3^, flip angle = 90°. All DICOM images of this study were managed by the eXtensible Neuroimaging Archive Toolkit (XNAT) [34].

## Results

In the first experimental setup we examined the B_1_^+^ transmit efficiency using a large cylindrical silicone oil phantom in a three-ring MRAS configuration. The magnitude of the B_1_^+^ field of the undistorted circularly polarized TE_11_ mode in the RF shield is displayed in Fig 3B. The image of the phantom acquired perpendicularly to the cylinder axis is slightly distorted. This is due to small deviations of in the linearity of the UHF gradient system. The B_1_^+^ transmit efficiency along the transversal plane of the cylindrical silicone oil phantom was determined by applying a turbo FLASH B_1_^+^ flip angle mapping sequence [33]. It was found to be B_1_^+^_Tx_eff_7T_ = 2.2 μT∙(kW)^-0.5^ for the MRAS, which is approximately twice that of the B_1_^+^ transmit efficiency of the conventional TW approach [24]. In the central region of interest (ROI) of about 10 cm diameter, the SNR = 101, which is nearly twice as large as that obtained in a standard 3 T Siemens Trio MRI body coil using the same phantom (SNR = 61) but just a quarter in comparison with the B_1_^+^ transmit efficiency (B_1_^+^_Tx_eff_3T_ = 8.4 μT∙(kW)^-0.5^).

When matter of low permittivity like air (ε_r_ = 1.0) or silicone oil (ε_r_ = 2.3) is present in the bore of the MRI system a undistorted fundamental TE_11_ mode is formed and the CP B_1_^+^ field typically possesses a high transversal field homogeneity within the central part of the ROI compromising about 10 cm diameter. Outside of this central part, the field homogeneity decreases due to the inherent transversal profile of the TE_11_ mode. During *in vivo* applications, however, there is a significant dielectric loading (ε_r_ = 50–80) in the MRI system bore that either distorts the TE_11_ mode or even leads to the excitation of higher order transverse magnetic (TM) modes [35].

The second experimental ring antenna setup with the water-NaCl-isopropanol phantom as load showed good imaging results (Fig 4B) in z-direction. The distortion is most pronounced at the borders of the water-NaCl-isopropanol phantom, which is caused by deviations from the linearity of the UHF gradient system. The B_1_^+^ transmit efficiency was determined along the coronal and transversal plane (Fig 4C and 4D) to yield B_1_^+^_Cor_Tx_eff_ = 1.48 μT∙(kW)^-0.5^ and B_1_^+^_Tra_Tx_eff_ = 1.95 μT∙(kW)^-0.5^. In the next step, the second experimental ring antenna setup was tested with *in vivo* measurements of an adult crab-eating macaque using the maximum VoI provided by the gradient system of the whole-body 7 T MRI system. Fig 5B shows the B_1_^+^ flip angle map covering the complete body of the monkey. The B_1_^+^ transmit efficiency was determined to B_1_^+^_Body_Tx_eff_ = 1.62 μT∙(kW)^-0.5^ inside the body and B_1_^+^_Head_Tx_eff_ = 0.72 μT∙(kW)^-0.5^ inside the monkey’s head. Fig 5C shows the anatomic MR images acquired with a FLASH sequence where the VoI extended about 45 cm along z-direction. However, the excitation is far from homogeneous with high signal intensities in the monkey close to both ring antennas. However, the central body parts of the monkey exhibited still sufficient B_1_^+^ excitation to provide a signal.

The 2D TSE sequence applied in the third experimental ring antenna setup required the maximum possible RF power and the highest B_1_^+^ transmit efficiency provided by the system. In order to maximize the SNR for neuroimaging applications, an optimally adapted 3-element phased array [36] primate head coil was used. The ring configuration in the third experimental setup led to an increase of the B_1_^+^ transmit efficiency to B_1_^+^_Tx_eff_ = 4 μT∙(kW)^-0.5^ inside the monkey’s brain tissue. The anatomic high-resolution image in Fig 6B displays both excellent contrast and homogeneity across the entire brain. Gray and white matter can be differentiated with great accuracy and the high-resolution allows a very detailed picture of the brain anatomy, which is most visible in the cerebellum, where even small structures are delineated with high precision. These results are comparable in quality to volume coil excitation approach results [37]. The Q-factor of metamaterial ring antennas for loaded and unloaded conditions in all experimental setups was only minimal varying with Q = [1.2,…,1.3].

## Discussion

In a theoretical study, the concept of MRAS was proposed [25] for UHF MRI, which combines a volume- selective B_1_^+^ field excitation in z-direction [26]. Simulations of the spatial distribution of the EM waves [38] showed that the MRAS is very versatile, as it provides highly confined field profiles appropriate, for instance, to larynx diagnostics, [26] while reducing the exposure of the irradiated RF energy. Thus, theoretical and numerical studies point to MRAS having a high potential for offering increased B_1_^+^ transmit efficiency at reduced exposure of the whole human body of the irradiated RF energy.

Here, we report on the first experimental realization of such a system, and studied its efficiency for imaging both small as well as whole-body volumes. Furthermore, the B_1_^+^ transmit efficiencies, the resolution, and the SNR of a metamaterial-based ring antenna system were investigated. The MRAS relies on the focused excitation of the fundamental transverse electric circular waveguide mode (TE_11_). The results show the possibility for selective excitation in z-plane for a research whole-body 7 T MRI system. Measurements of a silicone oil phantom (Fig 3B) indicate the presence of this mode, even if the image of the mode field within the phantom is slightly distorted due to small inhomogeneities in the static magnetic field of the MRI system. The distortions become more pronounced when the permittivity of the medium or subject increases, as seen when comparing the measurements of the silicone oil phantom (ε_r_ = 2.3) with the imaging data of the water-NaCl-isopropanol phantom and a crab-eating macaque.

The B_1_^+^ transmit efficiency of our MRAS was determined to B_1_^+^_Tx_eff_ = 2.2 μT∙(kW)^-0.5^ using the silicone oil phantom, a value comparable to that reported for travelling-wave approach [20]. In the cylindrical water-NaCl-isopropanol phantom, the B_1_^+^ transmit efficiency of the second experimental setup with only two ring antennas was minimal lower in transversal plane. This was caused by several effects: first, the inhomogeneous B_1_^+^ field distribution due to the higher permittivity (about 25 times higher than silicone oil) and electrical conductivity of water in a large volume and second by the dual propagation direction of the B_1_^+^ field when using this ring antenna configuration. Even the monkey as load does not lead to an increase of the B_1_^+^ transmit efficiency in the torso or in the head for this ring antenna setup. The third experimental ring antenna setup (Fig 6A) can increase the B_1_^+^ transmit efficiency to highest value of all measurements with B_1_^+^_Tx_eff_ = 4 μT∙(kW)^-0.5^ inside the monkey brain tissue. The main reason was the more focused B_1_^+^ field resulting from using one ring antenna as reflector. This according transmit efficiency measured in the 7 T MRI system is only around 2 times smaller than those obtained for standard clinical 3 T MRI systems (B_1_^+^_Tx_eff_ = 8.4 μT∙(kW)^-0.5^ measured at a 3 T Siemens Trio MRI body coil. Compared to other recent approaches destined to whole-body imaging, like coaxial waveguide MRI [23] or travelling-wave approach [20], the *in vivo* B_1_^+^ transmit efficiency of our MRAS is approximately twice as high.

The big advantage of the MRAS for whole-body imaging is its geometric and functional flexibility. On the one hand, MRAS offers the possibility to place the antennas flexibly and with ease at suitable positions, on the other hand, the functional behavior of each antenna can be chosen to act either act as transmitter, receiver, or reflector. Thus, the RF magnetic field distribution in the bore can be tailored using a particular arrangement of ring antennas according to the needs of each experiment. This freedom in the design of the MRAS was demonstrated here with three different experimental ring antenna setups (Figs 3A, 4A and 6A). This flexibility in the design makes the MRAS unique compared to any other experimental UHF MRI whole-body transmit concept [23,39–42], and allows a convenient imaging of the subject in the MR bore. For instance, in comparison to the conventional TW approach [20] the MRAS does not require any patch antenna for excitation. Such patch antennas may possibly block one side of the MRI bore thus potentially cause stress to patients due to the restricted space left in the bore.

The imaging studies using crab-eating macaques also demonstrated that high-resolution images could be acquired using an MRAS (Fig 5A) as well as its suitability for imaging of whole monkey (Fig 5C). Keeping in mind that the macaque fixation device occupied the central part of the MR bore (Fig 5A) and prevented the installation of any additional metamaterial ring antenna in this study. The high SNR enabled a resolution of 0.31×0.31×1.5 mm^3^, providing a very detailed view of the main parts of the crab-eating macaque brain. In fact, the gray and white matters, cerebellum and hippocampus are well delineated with a good contrast (Fig 6B). Even in low permittivity media like the silicone oil phantom the MRAS exhibited a high SNR, which is nearly twice as large as the SNR of a body coil inside a clinical 3 T MRI system.

The MRAS can also be applied for large volumes up to the limits of gradient system usually with 40 cm in z-direction and 40 cm in x-y direction. This was demonstrated by imaging a large silicone oil phantom and a water-NaCl-isopropanol phantom (with realistic material parameters of permittivity and conductivity) and the whole body of a macaque. In fact, a MRAS-equipped 7 T UHF MRI system opens up the possibility of imaging a maximum FoV. The reconstructed MR image (Figs 4B, 4D and 5C), however, shows non-homogenous illumination along the z-direction, i.e., the contrasts are weaker in the center of the image and brighter at its borders. Two factors contribute to this non-homogeneity, namely the distance of the ring antennas in terms of sensitivity to the phantom and subject, and the distribution of the B_1_^+^ field inside the water phantom and subject. The B_1_^+^ field homogeneity and B_1_^+^ transmit efficiency are given by the resulting mix of transverse electric and transverse magnetic modes, as well as the distribution of permittivities and conductivities. Nevertheless, the inhomogeneous B_1_^+^ field distribution is still an ongoing challenge at UHF MRI. To obtain a more homogeneous B_1_^+^ field distribution across the whole body, RF shimming [43,44] and application of dielectric pads [45] are suggested as promising complements.

In the present study the MRAS ability of acquiring data with high SNR from rather large VoIs was demonstrated using a silicone oil cylinder phantom (diameter = 40 cm) and a crab-eating macaque (length about 45 cm) as a load. The ring antennas installed inside the bore of the MRI system blocked any use of the patient table and prevents human subject tests. This shortcoming can be easily overcome by using metamaterial ring antennas whose inner diameters slightly exceed the 60 cm of the MRI bore. They could be integrated directly into the MRI system and placed behind its housing, such that the MRAS system no longer occupies a part of the volume of the bore, nor obstructs the operation of the moving table. Orzada et al. [46] demonstrated the possibility of installation of a 32-element transmit/receive body array for 7 T integrated between the bore liner and the gradient system. This body array uses a moving table and has 4 times higher RF power (32 kW). Thus, the combination of a MRAS and a moving table opens up the possibility of imaging the entire body of a human using a 7 T (or higher) MRI system [47]. The metamaterial ring antennas in all experimental setups didn't show strong load dependency of the Q-factor. This behavior is an advantage for applications as transmit system case but a disadvantage for application as receive system.

In conclusion, we have provided an experimental proof of concept [26,38] that UHF MRI systems (7 T or higher) equipped with a MRAS may be employed to measure VoIs of different sizes, including extended VoIs. The flexibility of the ring configuration and the ring functions opens up the opportunity for a tailor-made design of experimental setups, which then allow for imaging under a wide variety of conditions. Furthermore, the high SNR of MRAS-equipped MRI systems also suggest that such a system is well suited for high-resolution studies of small VoIs. Last but not least, the MRAS provides new options not only for proton but also for other heteronuclei (^19^F, ^23^Na) [48] due to its inherent, dual-band capability and good SNR for receive.

The outcome of the three experimental ring antenna setups leads us the conclusion that the Metamaterial Ring Antenna System should consists of a minimum number of five ring antennas to provide both high SNR and B_1_^+^ transmit efficiency. This system has to be fully integrated into the MRI system and should use two outer ring antennas as reflector, two ring antennas as transmitter and one central ring antenna for receive only.

As soon as 7 T UHF whole-body MRI systems obtain clinical approval, we expect that full-body MRAS-equipped MRI systems will improve the diagnostic potential of the UHF MRI when monitoring extended inflammation, searching for disseminated tumors and metastases, or analyzing systemic diseases. Ultimately, we believe that MRAS can boost the use of UHF whole-body MRI systems in clinical applications.

## Supporting information

S1 File B1 flip angle maps (DICOM format) of the water-NaCl-isopropanol phantom (transversal and coronal slices) and the crab-eating macaque (with sagittal slice). (Fig 4_Phantom_B1_cor.dcm; Fig 4_Phantom_B1_trans.dcm; Fig 5_monkey_b1_map.dcm).
(ZIP)

## References

[1] Bloch F, Hansen WW, Packard M. Nuclear induction. Phys Rev. 1946;70: 460–474.

[2] Purcell EM, Torrey HC, Pound RV. Resonance Absorption by Nuclear Magnetic Moments in a Solid. Phys Rev. 1946;69: 37–38. doi: 10.1103/PhysRev.69.37

[3] Lauterbur PC. Image Formation by Induced Local Interactions: Examples Employing Nuclear Magnetic Resonance. Nature. 1973;242: 190–191. doi: 10.1038/242190a0 2663289

[4] Kumar A, Welti D, Ernst RR. NMR Fourier zeugmatography. J Magn Reson 1969. 1975;18: 69–83. doi: 10.1016/0022-2364(75)90224-3 10.1016/j.jmr.2011.09.019 22152365

[5] Mansfield P, Pykett IL, Morris PG. Human whole body line-scan imaging by NMR. Br J Radiol. 1978;51: 921–922. doi: 10.1259/0007-1285-51-611-921 709046 10.1259/0007-1285-51-611-921

[6] Vaughan JT, Snyder CJ, DelaBarre LJ, Bolan PJ, Tian J, Bolinger L, et al 7 T Whole Body Imaging: Preliminary Results. Magn Reson Med. 2009;61: 244–248. doi: 10.1002/mrm.21751 19097214 10.1002/mrm.21751 PMC2875945

[7] Hayes CE, Edelstein WA, Schenck JF, Mueller OM, Eash M. An efficient, highly homogeneous radiofrequency coil for whole-body NMR imaging at 1.5 T. J Magn Reson 1969. 1985;63: 622–628. doi: 10.1016/0022-2364(85)90257-4

[8] Brink WM, Gulani V, Webb AG. Clinical applications of dual-channel transmit MRI: A review. J Magn Reson Imaging. 2015;42: 855–869. doi: 10.1002/jmri.24791 25854179 10.1002/jmri.24791

[9] Hayes CE. The development of the birdcage resonator: a historical perspective. NMR Biomed. 2009;22: 908–918. doi: 10.1002/nbm.1431 19856386 10.1002/nbm.1431

[10] Lauenstein TC, Freudenberg LS, Goehde SC, Ruehm SG, Goyen M, Bosk S, et al Whole-body MRI using a rolling table platform for the detection of bone metastases. Eur Radiol. 2002;12: 2091–2099. doi: 10.1007/s00330-002-1344-z 12136329 10.1007/s00330-002-1344-z

[11] Keupp J, Aldefeld B, Börnert P. Continuously moving table SENSE imaging. Magn Reson Med. 2005;53: 217–220. doi: 10.1002/mrm.20313 15690522 10.1002/mrm.20313

[12] Shajan G, Kozlov M, Hoffmann J, Turner R, Scheffler K, Pohmann R. A 16-channel dual-row transmit array in combination with a 31-element receive array for human brain imaging at 9.4 T. Magn Reson Med. 2014;71: 870–879. doi: 10.1002/mrm.24726 23483645 10.1002/mrm.24726

[13] Ogawa S, Lee TM, Kay AR, Tank DW. Brain magnetic resonance imaging with contrast dependent on blood oxygenation. Proc Natl Acad Sci U S A. 1990;87: 9868–9872. 2124706 10.1073/pnas.87.24.9868 PMC55275

[14] Goense JBM, Ku S-P, Merkle H, Tolias AS, Logothetis NK. fMRI of the temporal lobe of the awake monkey at 7 T. NeuroImage. 2008;39: 1081–1093. doi: 10.1016/j.neuroimage.2007.09.038 18024083 10.1016/j.neuroimage.2007.09.038

[15] Goluch S, Kuehne A, Meyerspeer M, Kriegl R, Schmid AI, Fiedler GB, et al A form-fitted three channel 31P, two channel 1H transceiver coil array for calf muscle studies at 7 T. Magn Reson Med. 2015;73: 2376–2389. doi: 10.1002/mrm.25339 25046817 10.1002/mrm.25339

[16] Hoult DI. Sensitivity and Power Deposition in a High-Field Imaging Experiment. J Magn Reson Imaging. 2000;12: 46–67. doi: 10.1002/1522-2586(200007)12:1<46::AID-JMRI6>3.0.CO;2-D 10931564 10.1002/1522-2586(200007)12:1<46::aid-jmri6>3.0.co;2-d

[17] Cho Z-H, Han J-Y, Hwang S-I, Kim D, Kim K-N, Kim N-B, et al Quantitative analysis of the hippocampus using images obtained from 7.0 T MRI. NeuroImage. 2010;49: 2134–2140. doi: 10.1016/j.neuroimage.2009.11.002 19909820 10.1016/j.neuroimage.2009.11.002

[18] Thalhammer C, Renz W, Winter L, Hezel F, Rieger J, Pfeiffer H, et al Two-Dimensional sixteen channel transmit/receive coil array for cardiac MRI at 7.0 T: Design, evaluation, and application. J Magn Reson Imaging. 2012;36: 847–857. doi: 10.1002/jmri.23724 22706727 10.1002/jmri.23724 PMC3445730

[19] Welsch GH, Juras V, Szomolanyi P, Mamisch TC, Baer P, Kronnerwetter C, et al Magnetic resonance imaging of the knee at 3 and 7 Tesla: a comparison using dedicated multi-channel coils and optimised 2D and 3D protocols. Eur Radiol. 2012;22: 1852–1859. doi: 10.1007/s00330-012-2450-1 22538628 10.1007/s00330-012-2450-1

[20] Brunner DO, Zanche ND, Fröhlich J, Paska J, Pruessmann KP. Travelling-wave nuclear magnetic resonance. Nature. 2009;457: 994–998. doi: 10.1038/nature07752 19225521 10.1038/nature07752

[21] Herrmann T, Mallow J, Plaumann M, Luchtmann M, Stadler J, Mylius J, et al The Travelling-Wave Primate System: A New Solution for Magnetic Resonance Imaging of Macaque Monkeys at 7 Tesla Ultra-High Field. PLoS ONE. 2015;10: e0129371 doi: 10.1371/journal.pone.0129371 26066653 10.1371/journal.pone.0129371 PMC4466239

[22] Brink WM, Webb AG. High permittivity pads reduce specific absorption rate, improve B1 homogeneity, and increase contrast-to-noise ratio for functional cardiac MRI at 3 T. Magn Reson Med. 2014;71: 1632–1640. doi: 10.1002/mrm.24778 23661547 10.1002/mrm.24778

[23] Alt S, Müller M, Umathum R, Bolz A, Bachert P, Semmler W, et al Coaxial waveguide MRI. Magn Reson Med. 2012;67: 1173–1182. doi: 10.1002/mrm.23069 22021117 10.1002/mrm.23069

[24] Mallow J, Herrmann T, Kim K-N, Stadler J, Mylius J, Brosch M, et al Ultra-high field MRI for primate imaging using the travelling-wave concept. Magn Reson Mater Phys Biol Med. 2013;26: 389–400. doi: 10.1007/s10334-012-0358-z 23233135 10.1007/s10334-012-0358-z

[25] Erni D, Liebig T, Rennings A, Koster NHL, Frohlich J. Highly adaptive RF excitation scheme based on conformal resonant CRLH metamaterial ring antennas for 7-Tesla traveling-wave magnetic resonance imaging. 2011 Annual International Conference of the IEEE Engineering in Medicine and Biology Society, EMBC. 2011. pp. 554–558. doi: 10.1109/IEMBS.2011.6090102 10.1109/IEMBS.2011.6090102 22254370

[26] Yang H, Liebig T, Rennings A, Froehlich J, Erni D. Tailored RF magnetic field distribution along the bore of a 7-Tesla traveling-wave magnetic resonance imaging system. 2013 International Conference on Electromagnetics in Advanced Applications (ICEAA). 2013. pp. 468–471. doi: 10.1109/ICEAA.2013.6632281

[27] Pendry JB, Schurig D, Smith DR. Controlling Electromagnetic Fields. Science. 2006;312: 1780–1782. doi: 10.1126/science.1125907 16728597 10.1126/science.1125907

[28] Constantinides CD, Atalar E, McVeigh ER. Signal-to-Noise Measurements in Magnitude Images from NMR Phased Arrays. Magn Reson Med. 1997;38: 852–857. 9358462 10.1002/mrm.1910380524 PMC2570034

[29] Dietrich O, Raya JG, Reeder SB, Ingrisch M, Reiser MF, Schoenberg SO. Influence of multichannel combination, parallel imaging and other reconstruction techniques on MRI noise characteristics. Magn Reson Imaging. 2008;26: 754–762. doi: 10.1016/j.mri.2008.02.001 18440746 10.1016/j.mri.2008.02.001

[30] Chen Z, Solbach K, Erni D, Rennings A. Electromagnetic Field Analysis of a Dipole Coil Element With Surface Impedance Characterized Shielding Plate for 7-T MRI. IEEE Trans Microw Theory Tech. 2016;64: 972–981. doi: 10.1109/TMTT.2016.2518168

[31] Gabriel S, Lau RW, Gabriel C. The dielectric properties of biological tissues: II. Measurements in the frequency range 10 Hz to 20 GHz. Phys Med Biol. 1996;41: 2251 doi: 10.1088/0031-9155/41/11/002 8938025 10.1088/0031-9155/41/11/002

[32] Brosch M, Budinger E, Scheich H. Different Synchronization Rules in Primary and Nonprimary Auditory Cortex of Monkeys. J Cogn Neurosci. 2013;25: 1517–1526. doi: 10.1162/jocn_a_00413 23647516 10.1162/jocn_a_00413

[33] Klose U. Mapping of the radio frequency magnetic field with a MR snapshot FLASH technique. Med Phys. 1992;19: 1099–1104. doi: 10.1118/1.596828 1518473 10.1118/1.596828

[34] Herrick R, Horton W, Olsen T, McKay M, Archie KA, Marcus DS. XNAT Central: Open sourcing imaging research data. NeuroImage. 2016;124, Part B: 1093–1096. doi: 10.1016/j.neuroimage.2015.06.076 26143202 10.1016/j.neuroimage.2015.06.076 PMC4965359

[35] Geschewski FH, Brenner D, Felder J, Jon Shah N. Optimum coupling and multimode excitation of traveling-waves in a whole-body 9.4T scanner. Magn Reson Med. 2013;69: 1805–1812. doi: 10.1002/mrm.24403 22782491 10.1002/mrm.24403

[36] Roemer PB, Edelstein WA, Hayes CE, Souza SP, Mueller OM. The NMR phased array. Magn Reson Med. 1990;16: 192–225. doi: 10.1002/mrm.1910160203 2266841 10.1002/mrm.1910160203

[37] Pfeuffer J, Merkle H, Beyerlein M, Steudel T, Logothetis NK. Anatomical and functional MR imaging in the macaque monkey using a vertical large-bore 7 Tesla setup. Magn Reson Imaging. 2004;22: 1343–1359. doi: 10.1016/j.mri.2004.10.004 15707785 10.1016/j.mri.2004.10.004

[38] Liebig T, Svedja JT, Yang H, Rennings A, Herrmann T, Mallow J, et al. Accurate and fast longitudinal RF magnetic field profiling for 7T traveling-wave MRI systems. Joint Annual Meeting ISMRM-ESMRMB 2014, Milano, Italy. 2014. p. 1358.

[39] Vaughan J t., Adriany G, Snyder C j., Tian J, Thiel T, Bolinger L, et al Efficient high-frequency body coil for high-field MRI. Magn Reson Med. 2004;52: 851–859. doi: 10.1002/mrm.20177 15389967 10.1002/mrm.20177

[40] Orzada S, Johst S, Maderwald S, Bitz AK, Solbach K, Ladd ME. Mitigation of B1+ inhomogeneity on single-channel transmit systems with TIAMO. Magn Reson Med. 2013;70: 290–294. doi: 10.1002/mrm.24453 22886695 10.1002/mrm.24453

[41] Ertürk MA, Raaijmakers AJE, Adriany G, Uğurbil K, Metzger GJ. A 16-channel combined loop-dipole transceiver array for 7 Tesla body MRI. Magn Reson Med. 2016; n/a-n/a. doi: 10.1002/mrm.26153 26887533 10.1002/mrm.26153 PMC4988950

[42] Raaijmakers AJE, Italiaander M, Voogt IJ, Luijten PR, Hoogduin JM, Klomp DWJ, et al The fractionated dipole antenna: A new antenna for body imaging at 7 Tesla. Magn Reson Med. 2016;75: 1366–1374. doi: 10.1002/mrm.25596 25939890 10.1002/mrm.25596

[43] Orzada S, Maderwald S, Poser BA, Bitz AK, Quick HH, Ladd ME. RF excitation using time interleaved acquisition of modes (TIAMO) to address B1 inhomogeneity in high-field MRI. Magn Reson Med. 2010;64: 327–333. doi: 10.1002/mrm.22527 20574991 10.1002/mrm.22527

[44] Brunner DO, Paška J, Froehlich J, Pruessmann KP. Traveling-wave RF shimming and parallel MRI. Magn Reson Med. 2011;66: 290–300. doi: 10.1002/mrm.22817 21695729 10.1002/mrm.22817

[45] Teeuwisse WM, Brink WM, Webb AG. Quantitative assessment of the effects of high-permittivity pads in 7 Tesla MRI of the brain. Magn Reson Med. 2012;67: 1285–1293. doi: 10.1002/mrm.23108 21826732 10.1002/mrm.23108

[46] Orzada S, Bitz AK, Kraff O, Oehmigen M, Gratz M, Johst S, et al. A 32-channel integrated body coil for 7 Tesla whole-body imaging. Singapore; 2016. p. 0167. Available: http://indexsmart.mirasmart.com/ISMRM2016/PDFfiles/0167.html

[47] Aldefeld B, Börnert P, Keupp J. Continuously moving table 3D MRI with lateral frequency-encoding direction. Magn Reson Med. 2006;55: 1210–1216. doi: 10.1002/mrm.20876 16598723 10.1002/mrm.20876

[48] Wolters M, Mohades SG, Hackeng TM, Post MJ, Kooi ME, Backes WH. Clinical Perspectives of Hybrid Proton-Fluorine Magnetic Resonance Imaging and Spectroscopy. Investig Radiol 5 2013 2013;48: 341–350. doi: 10.1097/RLI.0b013e318277528c 23211551 10.1097/RLI.0b013e318277528c

